# Editorial: Plastic Surgery for the Oncological Patient

**DOI:** 10.3389/fonc.2016.00191

**Published:** 2016-08-31

**Authors:** Adrien Daigeler

**Affiliations:** ^1^Department of Plastic Surgery, BG-University Hospital Bergmannsheil, Bochum, North Rhine-Westphalia, Germany

**Keywords:** plastic surgery, free flap, pedicled flap, breast reconstruction, soft-tissue sarcoma, soft-tissue defect, reconstruction in oncology, scalp defect

**The Editorial on the Research Topic**

**Plastic Surgery for the Oncological Patient**

Secondary to various surgical and non-surgical innovations for malignancy treatment, survival times associated with disease have improved for most cancer entities over the recent decades. Prolonged survival permits many oncologic patients to experience longer lives despite their advanced cancer stage. Working along the fellow cancer specialists, such as the oncologist, radiotherapist, and other surgical subspecialties, the plastic and reconstructive surgeon can add valuable tools to the armamentarium of oncological care in the palliative situation as well as importantly impact the cancer patient’s quality of life. Plastic surgery can help to cover defects after resection of ulcerating, bleeding, and fetid tumors. Painful ulcers can be excised and closed by tissue transfer. Large tumor masses causing pain or restrictions in function can be resected and defects covered. Patient care will be facilitated, and quality of life of the palliative patient will be improved, by allowing them to take part in social life for the time remaining.

Furthermore, plastic surgery techniques can be used to cover defects following curative tumor resection and, thereby, make resections possible that otherwise would not be compatible with life, such as large skull, thoracic, and abdominal wall resections. Tissue transfer allows for tension-free defect coverage and reduces wound complication rates and time to healing. Adjuvant radiation and chemotherapy can be administered early, and hospitalization is shorter. By augmenting the soft-tissue envelope, flap coverage can not only enable a timely start of radiation therapy but can also be used to mitigate its adverse effects by the transfer of healthy tissue in case of radiation ulcers. By tendon, nerve, or muscle transfers, reconstruction of motor function follows resection of nerves or important muscles. Vessels can be replaced by autologous or synthetic grafts. By a combination of these techniques, limb salvage is possible in most cases with malignancies at the extremities. In cases where limb salvage is not possible because of excessive tumor growth, advanced resection techniques like intra-thoraco-scapular amputations or hemipelvectomies may become necessary. Resulting defects can then be covered with remaining soft-tissue flaps from the amputated extremity. In situations where a complete resection of the tumor cannot be achieved, despite these techniques, it is even feasible to cover remaining tumor tissue with bulky flaps to delay ulceration.

Unfortunately, the oncologic plastic surgeon often faces the situation that patients are referred too late so that sufficient reconstruction is no longer possible, and ablative procedures need to be performed. Medical oncologists, radiotherapists, and other partners treating oncologic patients must be informed about the treatment options of plastic surgery. To spread the awareness of the possible values of plastic surgery techniques for the sake of the oncologic patient, several articles focusing on different areas of the body are combined in this ebook to give an overview.

The first article and section focuses on craniofacial oncology of scalp reconstruction. These patients are usually treated in cooperation with neurosurgeons who take care of the intracranial tumor and bony reconstruction, while the plastic surgeon takes care of the soft-tissue coverage. The latissimus dorsi flap eventually combined with a parascapular flap is a save option; however, recipient vessels for the flap must be identified carefully. The muscle part of the flap covered with split thickness skin graft results in an esthetical acceptable aspect and allows for wig placement.

The second series of articles deals with diverse options available for oncologic breast reconstruction as well as complex chest wall reconstruction in cancer.

Another article deals with the individualized breast reconstruction after mastectomy for breast cancer, including implant- and expander-based, flap-based, a combination of both, and breast reconstruction using fat grafting, giving the pros and cons for each technique. The following article refers to advanced stages of malignancies, making thoracic wall resections and reconstructions necessary. Possible reconstructional procedures are mentioned and weighed against the literature as well as information of the influence of thoracic wall resections on pulmonary function and quality of life is given.

The third section outlines the challenging aspects of truncal reconstruction, including the posterior aspect of the trunk, which challenges the plastic surgeon because of limited recipient vessels for free flap surgery and limited availability of large pedicled flaps. Therefore, an article illustrates alternative options ranging from vessel loops over combined perforator-based pedicled propeller flaps to free flaps anastomosed microsurgically to recipient perforator vessels at the back.

Further down the human body, the perineal region often requires defect coverage after extensive tumor resections. The corresponding article points out the value of the pedicled VRAM flap for perineal defects but also shows alternatives, including vessel loops for free flap reconstruction. In special cases, a two-stage procedure consisting of resection and vacuum dressing followed by definite coverage after confirmation of negative margins by the pathology report can be performed.

This strategy is also mentioned in the following articles of the fourth section dealing with reconstruction at the extremities. Although many studies have proven that the former dogma of wide excision can no longer be upheld in terms of recurrence and survival, complete resection should be achieved to reduce the local recurrence rate. In the extremities, functional structures, such as vessels and nerves, adjunct to the tumor should be preserved, but in some cases have to be resected with the tumor specimen. In these cases, functional reconstruction becomes necessary, which happens more often in the arm and especially the hand and foot than in the thigh or trunk because of the multitude of functionally relevant structures running in narrow spaces. In these cases, nerves, vessels, and muscles need to be replaced by microsurgical techniques or tendon tranfers perfomed. Additionally, free-tissue transfer is required in up to 70% after tumor resections to cover exposed bone, tendon or vessels. In special cases, even microsurgical bone transfer or prosthetic joint replacement is necessary. In these cases, co-operation with trauma or orthopedic surgeons is helpful to guarantee for the best possible functional outcome.

The last article and section discusses neoadjuvant and adjuvant treatment options, like radiation, chemotherapy, and isolated limb perfusion and their influence on plastic surgery techniques in terms of timing and complication rates.

While oncological safety remains of utmost importance, a multi-modal approach and advanced plastic surgery techniques can improve survival and quality of life as well as mitigate resulting functional deficits.

Due to the growing complexity of oncological care and the variety of disciplines involved, this can be best achieved in an inter-disciplinary setting of close collaborations (tumor boards, joint rounds, etc.) and is, therefore, well suited in specialized high-volume centers. The plastic and reconstruction surgeon should be an integral part to the combined multidisciplinary care of the oncologic patient.

## Author Contributions

The author confirms being the sole contributor of this work and approved it for publication.

## Conflict of Interest Statement

The author declares that the research was conducted in the absence of any commercial or financial relationships that could be construed as a potential conflict of interest. The reviewer I.L.V. and handling Editor declared their shared affiliation, and the handling Editor states that the process nevertheless met the standards of a fair and objective review.

